# Shaping transverse-tubules: central mechanisms that play a role in the cytosol zoning for muscle contraction

**DOI:** 10.1093/jb/mvad083

**Published:** 2023-10-17

**Authors:** Kohei Kawaguchi, Naonobu Fujita

**Affiliations:** Cell Biology Center, Institute of Innovative Research, Tokyo Institute of Technology, 4259 S2-11 Nagatsuta-cho, Midori-ku, Yokohama 226-8503, Japan; Cell Biology Center, Institute of Innovative Research, Tokyo Institute of Technology, 4259 S2-11 Nagatsuta-cho, Midori-ku, Yokohama 226-8503, Japan; Graduate School of Life Science and Technology, Tokyo Institute of Technology, 4259 S2-11 Nagatsuta-cho, Midori-ku, Yokohama 226-8503, Japan

**Keywords:** BIN1/Amph, dynamin, MTM, T-tubule, tubulation

## Abstract

A transverse-tubule (T-tubule) is an invagination of the plasma membrane penetrating deep into muscle cells. An extensive membrane network of T-tubules is crucial for rapid and synchronized signal transmission from the cell surface to the entire sarcoplasmic reticulum for Ca^2+^ release, leading to muscle contraction. T-tubules are also indispensable for the formation and positioning of other muscle organelles. Their structure and physiological roles are relatively well established; however, the mechanisms shaping T-tubules require further elucidation. Centronuclear myopathy (CNM), an inherited muscular disorder, accompanies structural defects in T-tubules. Membrane traffic-related genes, including MTM1 (Myotubularin 1), DNM2 (Dynamin 2), and BIN1 (Bridging Integrator-1), were identified as causative genes of CNM. In addition, causative genes for other muscle diseases are also reported to be involved in the formation and maintenance of T-tubules. This review summarizes current knowledge on the mechanisms of how T-tubule formation and maintenance is regulated.

## Graphical Abstract

**Graphical Abstract:**
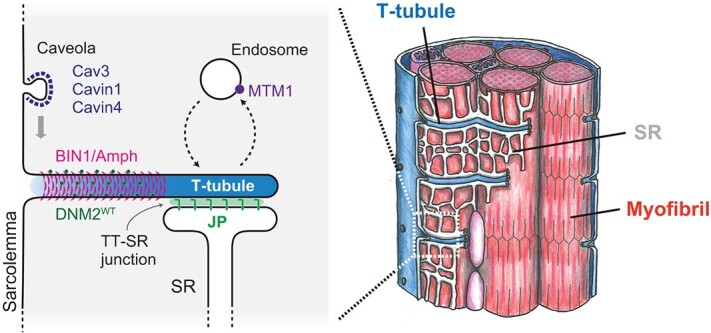

## Abbreviations

BAR: Bin/amphiphysin/Rvs
BIN1: Bridging Integrator-1
CNM: Centronuclear myopathy
CICR: calcium-induced calcium release
DHPR: dihydropyridine receptor
DNM2: Dynamin 2
EC: excitation-contraction
JP: Junctophilins
LGMD1C: limb-girdle muscular dystrophy
MTM1: Myotubularin 1
PI(3)P: phosphatidylinositol 3-phosphate
PI(5)P: phosphatidylinositol 5-phosphate
PI(3,5)P_2_: phosphatidylinositol 3,5-bisphosphate
PI: phosphoinositide
RyR: ryanodine receptor
SH3: Src homology 3 domain
SR: sarcoplasmic reticulum
XLMTM: X-linked myotubular myopathy

**Fig. 1:**
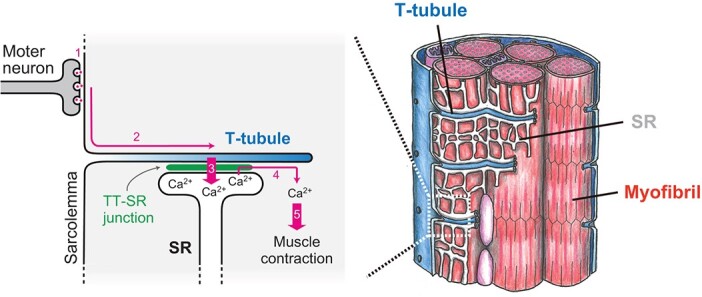
**T-tubule in excitation-contraction coupling.** Acetylcholine released from the motor neuron triggers depolarization of the sarcolemma (1). The electrical signal then propagates deep into the muscle cells via the T-tubule network (2). The signal is transmitted to the SR through the T-tubule-SR junctions and then activates RyR on the SR (3). This results in Ca2+ release from the SR to the cytoplasm (4). The increased Ca^2+^ concentration induces muscle contraction (5).

Muscles generate power for every movement in the animal body. Differentiated muscle cells are comprised of myofibrils, thousands of nuclei, and highly organized membrane structures, including Transverse-tubules (T-tubules) and sarcoplasmic reticulum (SR) (Fig. 1). Because muscle cells are huge, compartmentalization of cytosol by organelles, called cytosol zoning, is fundamental for synchronized contraction. A T-tubule is an invagination of the plasma membrane and an extensive membrane network penetrating deep into muscle cells. The SR is a specialized form of endoplasmic reticulum and surrounds the myofibrils in muscle cells. The contact sites between organelles are where the two structures physically interact, ensuring the proper positioning of organelles and muscle function. That is, T-tubules play a key role in the compartmentalization of cytosol in muscle cells, and the T-tubule-SR contact sites are indispensable for Ca^2+^ release from the SR for muscle contraction. This review will overview the mechanisms underlying the organization of the T-tubule system that play a central role in cytosol zoning for muscle function.

The history of T-tubule research is as follows: In 1881, Retzius hypothesized the existence of T-tubules because he noticed that the propagation of electrical depolarization within muscle cells was much faster than expected. Then, in 1897, Nyström observed that India ink entered rabbit heart muscle in a transverse pattern, providing the first structural clue about the T-tubule *(**1**)*. With advances in electron microscopy, in 1957, Porter and Palade successfully observed T-tubules, including the contact sites with the SR in rat skeletal muscle cells *(**2**)*. These historical studies show the existence and structure of the T-tubule system in muscle cells.

The primary function of T-tubules is to transmit signals instantaneously from the motor neuron to the interior of the muscle cell. Upon excitation–contraction coupling (EC coupling), acetylcholine released from a terminus of the motor neuron generates an action potential in the sarcolemma, the plasma membrane of muscle cells. This membrane potential travels the T-tubule system to quickly transmit the signal to the entire SR via T-tubule-SR contact sites. Then, Ca^2+^ is released from the SR for contraction (Fig. 1) *(**1**,**3**)*.

In vertebrate skeletal muscle, the voltage-gated calcium channel dihydropyridine receptor (DHPR) in the T-tubule membrane physically interacts with the ryanodine receptor (RyR), responsible for releasing Ca^2+^ from the SR membrane. The action potential induces a conformational change of DHPR, leading to the activation of the RyR receptor. As a result, Ca^2+^ is released from the SR into the cytosol. By contrast, in vertebrate cardiomyocytes and invertebrate muscle cells, it is the influx of extracellular Ca^2+^ mediated by DHPR that induces the activation of RyR. This process is called calcium-induced calcium release (CICR). Both mechanisms activate RyR and elevate Ca^2+^ levels in the cytosol of muscle cells *(**1**,**3**)*. The released Ca^2+^ then binds to the troponin complex, initiating a series of biochemical reactions leading to the sliding of actin and myosin filaments. In summary, T-tubules and their contact sites with the SR are crucial for rapid and synchronized signal transmission from the cell surface to the entire interior of muscle cells *(**1**,**3**)*.

## Hints from a Myopathy with T-Tubule Defects

Centronuclear myopathy (CNM), also referred to as myotubular myopathy, is a genetic disorder characterized by muscle weakness, atrophy, abnormal positioning of muscle cell nuclei, and a structural defect in the T-tubule. Unlike normal muscle cells, where nuclei are located at the periphery, CNM causes the nuclei to be centrally located within the muscle fiber. MTM1 (Myotubularin 1), DNM2 (Dynamin 2), and BIN1 (Bridging Integrator-1; also known as Amphiphysin 2) were identified as causative genes of CNM. Mutations in these genes disrupt the T-tubule membrane network. Other causative genes for muscle diseases are also reported to be involved in the formation and maintenance of T-tubules; however, their mechanisms have not been established *(**1**,**3**)*.

## BAR Domain Protein BIN1 Generates Curvature of T-Tubules

**Fig. 2:**
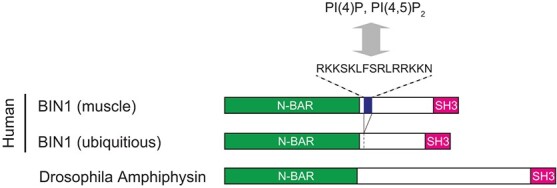
**Human and Drosophila Amphiphysin.** Amphiphysin contains the N-BAR domain and SH3 domain at the N-terminus and C-terminus, respectively. The skeletal muscle-specific splicing isoform of human BIN1 contains the PI-binding motif that interacts with PI(4)P and PI(4,5)P2. There is no PI-binding motif in Drosophila Amph.

BAR (Bin/amphiphysin/Rvs) domain proteins serve as lipid-binding, curvature-sensing, and membrane-bending proteins that regulate various membrane-related cellular processes, including endocytosis, phagocytosis, and cytokinesis *(**4**)*. The BAR domain superfamily is classified into three families: N-BAR, F-BAR, and I-BAR, which differentially recognize the degree and type of membrane curvature. One well-characterized BAR domain protein is Amphiphysin, which contains a N-BAR domain and a SH3 domain at its N-terminus and C-terminus, respectively (Fig. 2). The N-BAR domain forms a banana-shaped dimer with a positively charged concave face that binds to negatively charged phospholipids, generating membrane curvature. In contrast, the SH3 domain interacts with multiple proteins containing a proline-rich motif. Furthermore, the central region between the N-BAR and SH3 domains is an intrinsically disordered region containing a motif and domains including phosphoinositide (PI)-binding motif, clathrin and AP-2 binding domain, and myc-binding domain, which vary among isoforms and species *(**5**,**6**)*.

Humans encode two paralogues of Amphiphysin, namely Amphiphysin-1 and BIN1. Both Amphiphysin-1 and BIN1 play a key role in clathrin-mediated endocytosis by facilitating membrane curvature. Amphiphysin-1 and BIN1 were initially identified as brain-enriched adaptor proteins that recruit the membrane fission GTPase dynamin to facilitate the pinching off of clathrin-coated vesicles from the plasma membrane. This mechanism is crucial for the retrieval of components from synaptic vesicles *(**7*–*9**)*. Drosophila, an established model organism, has only one Amphiphysin isoform in its genome. Unexpectedly, Amphiphysin is dispensable for synaptic vesicle endocytosis in Drosophila. However, the mobility of flies is significantly affected by Amphiphysin null mutation. Razzaq *et al*. discovered that Amphiphysin was indispensable for T-tubule organization, EC coupling, and mobility *(**10**)*. Subsequent studies have revealed that BIN1 is critical for T-tubule formation in other animals, including mice *(**11**)*. Importantly, BIN1 was then identified as a causative gene for autosomal recessive CNM *(**12**)*. CNM patients with BIN1 mutation show abnormalities in T-tubules in skeletal muscle *(**12**,**13**)*. Consistent with this, homozygous mice with BIN1 null mutations, and those lacking the SH3 domain, exhibit postnatal lethality due to heart and skeletal muscle dysfunction *(**14**,**15**)*. Mice with skeletal muscle-specific deletion of BIN1 display a CNM phenotype with T-tubule abnormalities *(**16**)*. Furthermore, the expression level of BIN1 reaches its peak when the organized T-tubule is formed in mouse hearts *(**17**)*. Collectively, BIN1 is an evolutionarily conserved central player for T-tubule organization.

In human skeletal muscle, a muscle-specific splicing variant of BIN1 is expressed (Fig. 2). The splicing variant contains exon11 encoding a PI-binding motif in the central region *(**18**,**19**)*. This PI-binding motif shows a preference for PI(4)P and PI(4,5)P_2_. Although several studies showed the importance of the PI-binding motif, its role in T-tubule organization remains controversial. Mice lacking exon11 do not exhibit any muscle defects, whereas humans and dogs with mutations in the splice acceptor site of exon11 present with a CNM phenotype associated with T-tubule abnormalities *(**20**,**21**)*. In-cell tubulation assays also provided inconsistent results *(**17**,**22**)*, suggesting that the contribution of the PI-binding motif of exon11 for T-tubule organization would vary among species, tissues, and cell lines.

BIN1 alone is insufficient for the formation and maintenance of T-tubules. Interestingly, patients with CNM who have C-terminally truncated BIN1, lacking a functional SH3 domain, show T-tubule abnormalities *(**13**)*. This result suggests that the interactors with the SH3 domain play a critical role in T-tubule organization. Overexpressed BIN1-induced tubulation in cells depends on the expression of other genes associated with CNM and with other muscle diseases, including Cav3, MTM1, and DNM2 *(**17**,**22**,**23**)*. Therefore, these players should function coordinately with BIN1 in T-tubule organization.

## Dynamin2 Is Involved in T-Tubule Formation

**Fig. 3:**
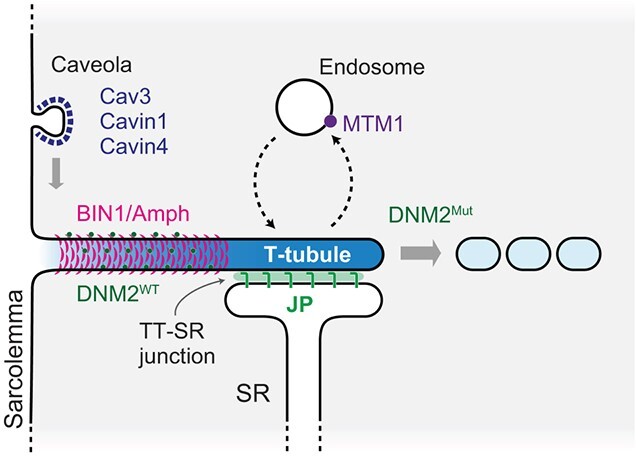
**Overview of mechanisms for T-tubule formation and maintenance.** Cav3, Cavin1, and Cavin4 regulate the initiation of T-tubule formation. BIN1/Amph and wild-type DNM2 are required for the formation of the T-tubule. JPs are involved in the formation of the T-tubule-SR junction and the maturation of the T-tubule. MTM1 plays a role in the maintenance of T-tubules by regulating endosomal membrane traffic.

Dynamin, a class of large GTPases, facilitates membrane fission in clathrin-mediated endocytosis. Humans have three dynamin genes: DNM1, DNM2, and DNM3. DNM1 and DNM3 are primarily expressed in the nervous system, while DNM2 is ubiquitously expressed in various tissues. Notably, DNM2 is implicated in autosomal dominant CNM. Hyperactive DNM2 mutations induce excess membrane fission, which results in the fragmentation of T-tubules (Fig. 3). Consistent with these findings, studies have demonstrated that the overexpression of wild-type or mutant DNM2 can induce the disorganization of T-tubules in mice, zebrafish, and Drosophila *(**15**,**24**,**25**)*. Thus, it is established that excess DNM2 activity disrupts the T-tubule membrane network.

The balance of the expression level of BIN1 and DNM2 is critical for the maintenance of T-tubules. In the developing heart of mice, the expression of BIN1 increases, correlating with T-tubule formation. Conversely, the expression of DNM2 decreases during this stage *(**17**)*. It was also reported that reduced DNM2 expression suppresses the postnatal lethality of mice lacking BIN1 *(**26**)*. Furthermore, increased BIN1 expression improved the T-tubule defect in mice carrying a CNM-associated DNM2 mutation *(**27**)*. These findings emphasize the crucial role of the balance between BIN1 and DNM2 in the formation and maintenance of T-tubules.

Basal DNM2 activity may contribute to T-tubule organization. The presence of DNM2 promotes BIN1-induced liposome tubulation *in vitro (**9**)*. Interestingly, the interaction between BIN1 and DNM2 blocks the GTPase activity of wild-type DNM2 but not the CNM-associated mutant. Fujise *et al*. demonstrated that the tubulation of the plasma membrane by overexpressed BIN1 depends on the co-expression of DNM2 and the interaction between DNM2 and BIN1 *(**23**)*. Therefore, it is thought that BIN1 and DNM2 function coordinately in T-tubule formation (Fig. 3).

## Caveolae Function in the Initiation Step of T-Tubule Formation

Caveolae are flask-shaped invaginations of the plasma membrane with a diameter of 50–100 nm, and play a crucial role in various cellular functions, including mechano-protection, vesicular trafficking, and signal transduction *(**28**)*. Unlike other regions of the plasma membrane, caveolae are enriched with cholesterol and sphingolipids. The major components of caveolae are caveolins, which are cholesterol-binding proteins. Cav1 and Cav3 are essential for caveolae formation among the caveolin family proteins in mammals. Cav1 is expressed in non-muscle cells, while Cav3 is specifically expressed in muscle cells *(**28**)*. Consistent with this, Cav1 expression decreases while Cav3 expression increases during the myotube formation of C2C12 cells *(**11**)*.

Cav3 is a causative gene for several muscle disorders. Specifically, mutations in Cav3 that lead to an approximately 90% reduction in Cav3 protein levels are associated with autosomal dominant limb-girdle muscular dystrophy (LGMD1C) *(**29**)*. LGMD1C patients and Cav3 null mice consistently exhibit the LGMD1C-like phenotype associated with T-tubule abnormalities *(**30**,**31**)*. Cav3 localizes to the sarcolemma and T-tubules in differentiated C2C12 myotubes *(**11**,**32**)*. Treatment with a cholesterol-binding drug Amphotericin B induces the redistribution of Cav3 and fragmentation of the T-tubule in differentiated C2C12 myotubes *(**33**)*. Furthermore, Cav3 is essential for membrane tubulation by BIN1 overexpression in primary human myotubes *(**22**)*. These findings indicate the importance of Cav3 in T-tubule organization.

Caveolin-associating coat proteins, known as cavins, are also involved in T-tubule organization. Among the four cavin proteins, Cavin1 (also known as PTRF) and Cavin4 (also known as MURC) have been reported to be involved in T-tubule formation. Cavin1 is expressed ubiquitously and is crucial for caveola formation by cooperating with Cav1 in non-muscle cells and with Cav3 in muscle cells *(**34**)*. Loss of Cavin1 results in T-tubule abnormalities in both mice and zebrafish *(**35**)*.

Cavin4 is one of the causative genes for dilated cardiomyopathy *(**36**)*. Cavin4 is expressed specifically in muscles, and its expression increases during the differentiation of C2C12 cells *(**37**)*. While Cavin4 is not essential for caveolae formation, it modulates caveolar morphology *(**34**)*. Notably, a recent study showed that Cavin4 bridges the caveola and BIN1. In zebrafish, which encode two paralogues, Cavin4a and Cavin4b, deficiency of both cavins leads to T-tubule abnormalities. Cavin4b localizes to the T-tubules through the interaction between its proline-rich motif to the SH3 domain of BIN1b *(**38**)*. Along with this study, another recent study reported the cooperation of caveolae and BIN1. In primary human myotubes, caveolae and BIN1 form a ring-like platform at the plasma membrane as a scaffold for initiating T-tubule formation *(**22**)*.

## Phosphoinositide 3-Phosphatase MTM1 Functions in T-Tubule Maintenance

MTM1, also known as Myotubularin 1, is a widely expressed phosphoinositide phosphatase that specifically removes the phosphate group at the third position. It dephosphorylates phosphatidylinositol 3-phosphate (PI(3)P) and phosphatidylinositol 3,5-bisphosphate (PI(3,5)P_2_) to PI and phosphatidylinositol 5-phosphate (PI(5)P), respectively *(**39**)*. PI(3)P and PI(3,5)P_2_ regulate endosomal and lysosomal functions through recruiting effector proteins, such as EEA1, Vps27/Hrs, and PROPPINs *(**39**)*. MTM1 is the most common causative gene for X-linked myotubular myopathy (XLMTM), characterized by progressive muscle atrophy and centronuclear abnormalities *(**40**)*. MTM1 knockout mice phenocopy XLMTM pathology, including T-tubule abnormalities. Importantly, MTM1 knockout mice exhibit a milder T-tubule defect at the postnatal stage than in later stages, suggesting that MTM1 is primarily involved in maintaining T-tubules rather than their formation *(**41**)*. Consistent with this, MTM1 is indispensable for T-tubule remodeling/maintenance but not for T-tubule formation in Drosophila and Zebrafish *(**42**,**43**)*. The precise mechanism as to how MTM1 maintains T-tubules remains to be addressed. Further studies are also necessary to reveal its mechanisms and implications in XLMTM pathology.

## Junctophilins Play a Critical Role in EC Coupling and T-Tubule Organization

Junctophilins (JPs) are a family of proteins that connect the endoplasmic reticulum and plasma membrane. Among the four JPs, JP1 and JP2 connect the T-tubule and the SR (Fig. 3). Their C-terminal transmembrane domain anchors to the SR, while their N-terminal region contains MORN motifs that interact with the T-tubule. JP1 is expressed at a high level in skeletal muscle and a low level in the heart. On the other hand, JP2 is expressed in both skeletal muscle and the heart, and it is the predominant isoform in the heart. Mice lacking both JP1 and JP2 show abnormalities in the triads and dyads in skeletal and heart muscle, respectively, show postnatal lethality, as well as defects in EC coupling. JP1 and JP2 physically bridge the T-tubule and terminal cisternae from the SR, facilitating EC coupling in skeletal and heart muscles *(**44**,**45**)*. JP2 also plays a critical role in T-tubule maturation and maintenance in the heart. Studies with mice with a cardiac-specific knockdown of JP2 exhibited heart dysfunction associated with T-tubule abnormalities *(**46**,**47**)*, indicating that anchoring the T-tubule with the SR is essential for proper T-tubule formation. Thus, JP is required for both T-tubule-SR junction formation and proper T-tubule maturation.

## Conclusion

In this review we summarized the molecular mechanisms of the formation and maintenance of T-tubules in muscle cells. Most T-tubule regulators have been identified through forward genetics based on human hereditary myopathies. Then these regulators are frequently functionally analyzed in mice and cultured cell systems. Our knowledge of the mechanisms of T-tubule organization is still far from a comprehensive understanding.

Revisiting the current model of T-tubule formation, a recent study shows that intrinsically disordered proteins generate membrane curvature in addition to BAR domain proteins *(**48**)*. It has also been reported that highly multimerized F-BAR domain proteins require scaffold proteins that interact with the F-BAR SH3 domain *(**49**)*. Furthermore, studies have shown that liquid–liquid phase separation occurs on membranes, providing a platform for various biological processes *(**50**)*. Particularly, N-BAR protein endophilin drives phase separation through multivalent interaction between its SH3 domain and the proline-rich motifs of accessory proteins, which may stabilize membrane curvature *(**51**)*. These findings collectively imply the role of protein condensates in T-tubule formation. Thus, the interplay among BAR domain proteins, intrinsically disordered proteins, and scaffold proteins might play a role in shaping and organizing the T-tubule structure.

More reverse genetics approaches are required to understand the mechanisms shaping T-tubules comprehensively. Genetically tractable model organisms such as zebrafish and Drosophila are suitable for these approaches. For example, in a recent study by Parton’s group, *in vivo* knockdown and overexpression screening in zebrafish revealed that the endocytic machinery is linked to T-tubule organization *(**52**)*. This study demonstrates that zebrafish is an amenable model for reverse genetic approaches in T-tubule research. Furthermore, Drosophila is also a suitable model organism for T-tubule study, as shown in the seminal work in which Amphiphysin was identified as a T-tubule shaping protein for the first time *(**10**)*. Interestingly, the T-tubule is not essential for the viability of Drosophila, probably because fly muscle cells are relatively thin. Ca^2+^ influx from the extracellular space may induce muscle contraction, albeit at a lower efficiency. Proteomics of T-tubules has been impossible until recently because the T-tubule membrane network cannot be isolated by conventional subcellular fractionation methods. Proximity labeling does not require fractionation for organelle proteomics, and it is relatively easy in Drosophila *(**53**)*; therefore, proteomics of T-tubule is now feasible. Furthermore, T-tubules could be observed through the cuticle by confocal microscopy in live Drosophila expressing GFP-tagged marker protein *(**54**)*. Combining proximity proteomics and in vivo RNAi screening in Drosophila would enable us to identify new essential T-tubule-related genes.
